# Mineral Content of the Pulp and Peel of Various Citrus Fruit Cultivars

**DOI:** 10.1007/s12011-019-01727-1

**Published:** 2019-04-27

**Authors:** Anna Czech, Ewa Zarycka, Dmytro Yanovych, Zvenyslava Zasadna, Izabela Grzegorczyk, Sylwia Kłys

**Affiliations:** 1 Department of Biochemistry and Toxicology, Faculty of Biology and Animal Production, University of Life Sciences in Lublin, Akademicka 13, 20-950 Lublin, Poland; 2 Instrumental Methods of Control Laboratory, State Scientific-Research Control Institute of Veterinary Medicinal Products and Feed Additives, Lviv, Ukraine

**Keywords:** Citrus fruits, Peel, Pulp, Minerals

## Abstract

The aim of the study was to compare the mineral content between the peel and the pulp of citrus fruits and to determine which citrus fruit, among orange (*Citrus sinensis*), pomelo (*Citrus maxima*), mandarin (*Citrus reticulata* Blanco), lemon (*Citrus limon*), key lime (*Citrus aurantifolia*), and red, yellow, or green grapefruit (*Citrus paradisi*), is the richest in minerals. The research material consisted of fresh citrus fruits belonging to the genus *Citrus* L in the family Rutaceae. The fruits were purchased at a supermarket at one time. To prepare laboratory samples, each fruit was cut in half, and one half was homogenized, treating the sample as a whole (peel + flesh), while the other half was peeled and the pulp (F) and peel (P) were homogenized separately. To determine the content of minerals (Na^+^, K^+^, Ca^+2^, Mg^+2^, Fe^+2^, Zn^+2^, Cu^+2^, Mn^+2^, and Se^+2^), the samples were mineralized and analyzed using an Analytik Jena PlasmaQuant PQ 9000 inductively coupled plasma optical emission spectrometer. The content of macro- and micronutrients in the peel of most of the fruits far exceeded their quantity in the pulp. Oranges and pomelos are the fruits richest in iron and copper, so they could be recommended in cases such as hemoglobin production disorders resulting from a deficiency of these elements. Oranges can additionally enrich the body with potassium, phosphorus, and manganese, while lime can be a source of calcium, zinc, sodium, and especially potassium. It should also be noted that all citrus fruits are a very valuable source of potassium, which is needed to ensure the water and electrolyte balance.

## Introduction

Citrus fruits (and products derived from them) are among the most commonly consumed fruits in the world, and their production remains high [1]. They include oranges, lemons, grapefruits, limes, mandarins, pomelos, kumquats, tangelos, and others. Due to their flavor and aroma, they are widely used in the food, cosmetics, and pharmaceutical industries [2]. They owe their popularity in part to their diversity of flavor; they can be sweet (oranges and mandarins) or sour (lemons, limes, and grapefruit). In the food industry, they are found in the form of raw fruit or as semi-finished products, such as must, pulp, puree, or frozen fruit. Essential oils obtained from these fruits are used in the cosmetics industry, and biologically active substances isolated from the peel or pulp are used in the pharmaceutical industry [3, 4].

Citrus fruits, due to the presence of many nutrients, such as vitamins A and E, B vitamins (thiamine, riboflavin and niacin), minerals, and antioxidants, i.e., flavonoids, vitamin C, phenolic compounds, and carotenoids, as well as dietary fiber, have a positive stimulatory effect on the immune, cardiovascular, and digestive systems [4]. They also have anti-inflammatory, anti-sclerotic, antiviral, antibacterial, and anti-cancer properties [5–7].

The content of valuable bioactive substances varies in individual parts of citrus fruits. The undervalued peel contains a wide variety of secondary components with high antioxidant activity relative to other parts of the fruit. It is also a valuable source of molasses, pectin, and limonene [4, 8]. Despite its high nutritional value, the rind is usually dried, mixed with dried pulp and used as cattle feed [2, 9], or disposed of and treated as waste, which constitutes 50% of the original weight of the fruit [6, 10]. This is considered a potential source of environmental pollution [11].

In addition, the peel of citrus fruit, like the pulp, contains many natural prenyloxycoumarin compounds, such as auraptene, bergamottin, imperatorin, and heraclenin, as well as macro- and micro-minerals, whose presence increases its dietary and therapeutic value [12]. Therefore, the low cost and availability of the peel, which is a waste product of citrus fruit, can be considered a potential source of nutraceuticals [4]. Citrus peels, being rich in bioactive compounds, can be used to produce functional foods or natural “dietary supplements”, providing not only dietary fiber and antioxidant compounds, but also minerals.

Trace element levels in fruit may be influenced by the mineral composition of the soil on which it was grown, the composition of the irrigation water, weather conditions, and agricultural practices, such as the types and amounts of fertilizers used. The fruit variety also has a significant impact on mineral content [13, 14]. Fruits selectively accumulate trace metals. Pineapple, for example, shows a high level of manganese compared to other fruits [15].

Although the citrus pulp is recognized as providing a source of some mineral elements such as potassium, calcium phosphorus, or magnesium for human nutrition, there are other parts of the fruit which also contain these elements. These other parts of the fruit are not recognized in nutrition because they are generally the non-edible components, like peels. According to Barros et al. [11], the orange, the lime, and the mandarin peels, alike in their pulps, are promising sources of mineral elements which can be used for their health properties in food products. They can be applied as a source of functional compounds [11]. At present, little is known about the levels of trace elements in citrus fruits and their parts such as peel or pulp [13].

The aim of the study was to compare the mineral content between the peel and pulp of citrus fruits and to determine which citrus fruit, among orange (*Citrus sinensis*), pomelo (*Citrus maxima*), mandarin (*Citrus reticulata* Blanco), lemon (*Citrus limon*), key lime (*Citrus aurantifolia*), and red, yellow, or green grapefruit (*Citrus paradisi*), is the richest in minerals.

## Material and Methods

### Samples

The research material consisted of fresh citrus fruits belonging to the genus *Citrus* L in the family Rutaceae. The following species of citrus fruit were analyzed:

- Orange (*Citrus sinensis*), Navelina cultivar (Turkey)
- Mandarin (*Citrus reticulata* Blanco), Clementina cultivar (Turkey)
- Lemon (*Citrus limon*), Interdonato cultivar (Turkey)
- Key lime (*Citrus aurantifolia*), Tahiti cultivar (Turkey)
- Pomelo (*Citrus maxima*), Honey cultivar (Israel – the only country supplying this fruit to the Polish market)
- Red grapefruit (*Citrus paradisi*), Star Ruby cultivar (Turkey)
- Yellow grapefruit (*Citrus paradisi*), Duncan cultivar (Turkey)
- Green grapefruit (*Citrus paradisi*), Sweetie cultivar (Turkey)

The fruits were purchased at a supermarket at one time.

### Preparation and Mineralization of Samples

Primary samples of each fruit were taken from three different packets from one supplier. Three fruits were taken randomly from each packet. In total, 72 fruit pieces were used for the research (3 fruits × 3 packets × 8 species).

To prepare laboratory samples, each fruit was cut in half and one half was homogenized, treating the sample as a whole (peel + pulp), while the other half was peeled and the pulp (F) and peel (P) were homogenized separately. These steps were performed immediately after the fruit had been purchased. Prior to homogenization, each fruit was washed separately underwater (about 60–70 °C) and dried with a paper towel in order to remove impurities that could affect the assay result. The homogenized samples were divided into two parts. One part was used for moisture analysis. Moisture in the samples was determined by drying 5 g of the samples at 105 °C to a constant weight [16]. The other portion was placed in plastic flasks and kept deep-frozen at − 80 °C until analysis. The samples were homogenized using the BUCHI mixer B-400 with ceramic knives.

Mineralization was carried out in a Microwave Digestion System (Milestone Start D) in Teflon vials. The program of temperature changes in autoclaves during mineralization is presented in Table 1.

**Table 1:** Parameters of temperature changes during mineralization in autoclaves

Stage	Time (min. sec.)	Temperature (°C)	Radiation power (W)
1	5.00	80	< 350
2	3.30	160	< 800
3	4.30	190	< 1000
4	12.00	190	< 800

The pressure throughout the mineralization process did not exceed 12 bar (1.2 MPa). A clear solution was obtained when the mineralization process was completed. Then the solution was cooled to room temperature and transferred to a 25 mL volumetric flask, which was filled with demineralized water (ELGA Pure Lab Classic).

All glassware was rinsed with tap water, soaked in an acid bath (5 M HNO_3_) for 24 h, rinsed with demineralized water, and dried before use, in order to minimize the risk of metal contamination. A 6 mL volume of concentrated HNO_3_ (Fluka, France) was poured overweighed portions (6.0 ± 0.1 g of each sample).

### Chemicals and Solutions

All reagents were of analytical grade. In the case of Na and K determinations, cesium chloride (Merck, Poland) was added to the standards and samples as an ionization buffer at a concentration of 0.2% *w*/*v*. Ca and Mg were analyzed by adding 0.4% *w*/*v* lanthanum oxide (Merck, Poland), a correction buffer that enables binding of the analyzed element to the matrix.

Standard solutions of Fe at a concentration of 20 ± 1 mg L^−1^, Cu at 50 ± 2 mg L^−1^, Mn at 20 ± 1 mg L^−1^, Se at 100 ± 2 mg L^−1^ (Certipur®Certified Reference Material, Merck, Darmstadt, Germany), and Zn standard for ICP (TraceCERT®, Sigma Aldrich) at 1000 ± 2 mg L^−1^ were used. A multi-element intermediate solution was prepared by means of sequential dilutions with the abovementioned elements in 5% (*v*/*v*) HNO_3_ (diluted from concentrated nitric acid, Fluka, France) and used to obtain calibration curves.

Demineralized water from ELGA Pure Lab Classic was used to prepare all solutions.

### Instrumentation and Measurement

Na and K were analyzed using flame atomic emission spectroscopy (FAES) with a flame photometer (Pye Unicam SP 2900, Cambridge, UK) at wavelengths of *λ* = 589.0 and *λ* = 766.5 nm, respectively. Measurements of elements (Ca, Mg, Fe, Zn, Cu, Mn, Se) were performed using an Analytik Jena PlasmaQuant PQ 9000 inductively coupled plasma optical emission spectrometer. The operational conditions, analytical lines, and wavelengths of the elements were as follows: RF generator power, 1200 W; RF generator frequency, 40.68 MHz; coolant gas flow rate, 12 L min^−1^; carrier gas flow rate, 0.5 L min^−1^; auxiliary gas flow rate, 0.6 L min^−1^ max. Integration time, 15 s; pump rate, sample injection 19 rpm at normal mode (1 mL min^−1^); and flush fluid injection 78 rpm at fast mode (4 mL min^−1^); viewing configuration, axial; replicates, 3; flush time, 30 s; wavelengths of absorption (resonance) lines (nm), 422.7 for calcium; 285.2 for magnesium; 238.2 for iron; 206.2 for zinc; 327.4 for copper; 257.6 for manganese; and 196.0 for selenium. The phosphorus content was determined by spectrometry at 400 nm using a Helios Alpha UV-VIS apparatus (Spectronic Unicam, Leeds, UK), according to AOAC [16].

The methods were validated in accordance with the requirements of the Commission Decision 2002/657/EC. All sample solutions were prepared using an amount of multi-element standard that corresponded to the mean values of the calibration curve. The solutions were analyzed under optimized conditions.

### Statistical Analysis

Statistical data analysis was carried out using the commercial program Statistica, version 13.3.

Tables 2 and 3 present the results of the two-way analysis of variance (ANOVA). The mathematical model takes into account the influence of the fixed factors S—the fruit species (orange, pomelo, mandarin, etc.) and P—the part of the fruit (pulp or peel)—on the content of mineral elements in the sample.

$$ {\mathrm{Y}}_{\mathrm{i}\mathrm{jk}}=\upmu +{\mathrm{S}}_{\mathrm{i}}+{\mathrm{P}}_{\mathrm{j}}+{\mathrm{E}}_{\mathrm{i}\mathrm{jk}} $$

μ: characteristics of average
S_i_: fixed effect of the fruit species
P_j_: fixed effect of the part of the fruit
E_ijk_: measurement error

**Table 2:** Content of macronutrients in the pulp (F) and peel (P) of citrus fruits

Fruit/index		Orange	Pomelo	Mandarin	Lemon	Key lime	Red grapefruit	Green grapefruit	White grapefruit	%RDS
Moisture %	F	90.1^a^	83.3^c^	88.2^b^	89.0^b^	89.2^a^	92.3^a^	91.9^a^	90.0^a^	12.8
	P	68.1^b^	78.0^a^	77.7^a^	78.1^a^	70.3^b^	80.4^a^	80.2^a^	79.9^a^	15.6
	*P*_value_	0.03	0.02	0.03	0.04	0.03	0.04	0.03	0.04	
Potassium mg 100 g^−1^	F	139^a^	104^def^	133^ab^	113^ce^	145^a^	111^cf^	123^bc^	117^cd^	11.9
	P	154^a^	127^def^	141^bc^	127^def^	152^ab^	132^ce^	133^cd^	129^cf^	9.33
	*P*_value_	0.06	< 0.01	0.08	0.06	0.223	< 0.01	0.05	0.08	
Sodium mg 100 g^−1^	F	0.12^d^	0.10^d^	1.11^c^	1.89^b^	3.10^a^	0.12^d^	0.21^d^	0.16^d^	125
	P	0.54^d^	0.68^d^	1.09^c^	1.99^b^	3.88^a^	0.25^e^	0.31^e^	0.25^e^	108
	*P*_value_	< 0.01	< 0.01	0.48	0.07	0.04	< 0.01	0.02	0.02	
Calcium mg 100 g^−1^	F	27.9^b^	14.5^d^	24.9^b^	18.0^cd^	41.3^a^	21.3^c^	24.5^b^	22.6^bc^	32.9
	P	41.9^b^	28.8^e^	37.1^bc^	31.8^de^	63.9^a^	36.0^c^	38.9^b^	34.8^cd^	36.5
	*P*_value_	0.03	< 0.01	0.01	0.04	< 0.01	0.03	0.04	0.03	
Phosphorus mg 100 g^−1^	F	23.3^a^	18.9^ab^	18.7^ab^	18.0^ab^	17.9^ab^	15.1^b^	19.0^ab^	17.0^ab^	11.7
	P	25.3^a^	21.9^a^	19.9^a^	23.9^a^	20.1^a^	20.0^a^	22.5^a^	19.0^a^	16.6
	*P*_value_	0.40	0.27	0.56	0.02	0.23	0.05	0.14	0.15	
Magnesium mg 100 g^−1^	F	10.3^bc^	19.40^a^	10.4^bc^	8.40^cd^	11.6^b^	8.07^cd^	7.99^d^	9.00^cd^	34.6
	P	13.2^b^	23.0^a^	12.9^b^	11.50^b^	13.0^b^	10.0^b^	9.50^b^	11.1^b^	35.2
	*P*_value_	0.04	0.03	0.06	0.04	0.36	0.02	0.23	0.04	

*F* pulp, *P* peel

^a^, ^b^, ^c^, ^d^, ^e^, ^f^Values in rows with different letters are significantly different at *p* ≤ 0.05: F rows, superscript letters indicate statistically significant differences in mineral content in the pulp (F) between citrus cultivars; P rows, superscript letters indicate statistically significant differences in mineral content in the peel (P) between citrus cultivars. The results of two-way analysis of variance (ANOVA)

*P* value, level of significance of differences between pulp (F) and peel (P) of individual citrus fruits

**Table 3:** Content of micronutrients in the pulp (F) and peel (P) of citrus fruits

Fruit/index		Orange	Pomelo	Mandarin	Lemon	Key lime	Red grapefruit	Green grapefruit	White grapefruit	%RDS
Iron mg 100 g^−1^	F	0.37^b^	0.46^a^	0.24^cde^	0.28^d^	0.21^cde^	0.19^e^	0.21^cde^	0.20^e^	35.6
	P	0.51^a^	0.52^a^	0.33^d^	0.34^d^	0.41^b^	0.23^f^	0.27^e^	0.28^e^	32.6
	*P*_value_	< 0.01	< 0.01	< 0.01	< 0.01	< 0.01	< 0.01	< 0.01	0.02	
Zinc mg 100 g^−1^	F	0.17^b^	0.10^c^	0.23^ab^	0.17^b^	0.24^a^	0.17^b^	0.22^ab^	0.19^ab^	27.2
	S	0.25^a^	0.12^b^	0.29^a^	0.28^a^	0.26^a^	0.33^a^	0.30^a^	0.28^a^	28.2
	P_value_	0.01	0.10	0.05	< 0.01	0.50	< 0.01	0.03	0.01	
Copper mg 100 g^−1^	F	0.06^a^	0.05^b^	0.04^c^	0.04^cd^	0.04^c^	0.03^d^	0.05^b^	0.05^b^	20.3
	P	0.15^a^	0.21^a^	0.05^de^	0.04^e^	0.06^cd^	0.08^b^	0.08^b^	0.07^bc^	67.3
	*P*_value_	< 0.01	< 0.01	< 0.01	0.07	< 0.01	< 0.01	< 0.01	< 0.01	
Manganese mg 100 g^−1^	F	0.02^b^	0.01^b^	0.02^b^	0.06^a^	0.01^b^	0.01^b^	0.01^b^	0.02^b^	112
	P	0.13^a^	0.15^a^	0.12^ab^	0.05^de^	0.04^e^	0.10^bc^	0.10^c^	0.07^d^	52.1
	*P*_value_	< 0.01	< 0.01	< 0.01	0.78	< 0.01	< 0.01	< 0.01	< 0.01	
Selenium μg 100 g^−1^	F	1.50^bcd^	1.30^cd^	1.70^abc^	1.78^ab^	1.98^a^	1.28^d^	2.05^a^	1.79^ab^	18.9
	P	2.35^bc^	2.00^bc^	3.38^abc^	4.13^a^	3.10^abc^	1.68^c^	3.55^ab^	2.28^bc^	38.5
	*P*_value_	< 0.01	0.02	0.03	< 0.01	< 0.01	< 0.01	< 0.01	< 0.01	

Legend: see Table 2

Table 4 presents the results of one-way analysis of variance (ANOVA) used to determine the fixed effect of the species of fruit—S (orange, pomelo, mandarin, etc.) on the homogenate content of the fruit on the mineral elements content in the sample a was used.

$$ {\mathrm{Y}}_{\mathrm{i}\mathrm{j}}=\upmu +{\mathrm{S}}_{\mathrm{i}}+{\mathrm{E}}_{\mathrm{i}\mathrm{j}} $$

μ: characteristics of average
S_i_: fixed effect of the fruit species
P_j_: fixed effect of the part of the fruit
E_ijk_: measurement error

**Table 4:** Content of micro- and macro-elements in whole citrus fruits

Fruit/index		Orange	Pomelo	Mandarin	Lemon	Key lime	Red grapefruit	Green grapefruit	White grapefruit	%RDS
Potassium	mg 100 g^−1^	145^a^	117^b^	133^ab^	120^b^	147^a^	120^b^	126^ab^	124^ab^	11.3
Sodium	mg 100 g^−1^	0.36^d^	0.38^d^	1.19^c^	1.86^b^	3.41^a^	0.16^e^	0.26^e^	0.21^e^	115
Calcium	mg 100 g^−1^	34.0^b^	20.0^c^	30.1^b^	25.9^c^	51.0^a^	27.4^b^	30.0^b^	26.2^b^	35.7
Phosphorus	mg 100 g^−1^	24.1^a^	21.1^b^	17.9^b^	21.8^b^	19.0^b^	21.2^b^	22.0^b^	17.0^b^	18.0
Magnesium	mg 100 g^−1^	10.5^b^	21.9^a^	11.1^b^	9.86^b^	12.2^b^	9.99^b^	8.98^b^	11.1^b^	35.7
Iron	mg 100 g^−1^	0.45^a^	0.49^a^	0.29^b^	0.31^b^	0.31^b^	0.21^c^	0.24^bc^	0.24^bc^	34.8
Zinc	mg 100 g^−1^	0.21^b^	0.11^c^	0.26^a^	0.22^ab^	0.24^a^	0.25^a^	0.26^a^	0.23^ab^	31.4
Copper	mg 100 g^−1^	0.11^a^	0.12^a^	0.04^c^	0.04^c^	0.04^c^	0.06^b^	0.07^b^	0.07^b^	64.4
Manganese	mg 100 g^−1^	0.08^a^	0.08^a^	0.07^ab^	0.05^bc^	0.03^d^	0.06^b^	0.06^b^	0.04^c^	86.9
Selenium	μg 100 g^−1^	1.80^d^	1.61^e^	2.58^b^	2.77^a^	2.53^b^	1.48^f^	2.79^a^	2.13^c^	42.4

^a^, ^b^, ^c^, ^d^, ^e^, ^f^Values in rows with different letters are significantly different at *p* ≤ 0.05. The results of one-way analysis of variance (ANOVA)

Significance of differences between means was determined using the Duncan test, assuming a significance level *p* = 0.05 and *p* = 0.01.

## Results and Discussion

### Mineral Elements in Pulp and Peel of Citrus Fruits

Citrus fruits are a rich source of potassium, with one orange assumed to provide 6% of the dietary reference intake (DRI), while one glass of orange juice provides 10% of the DRI [17]. This element is involved in the regulation of the water and electrolyte balance and the acid-base balance in the body [18, 19]. Our study indicated that the potassium content was similar in the pulp and peel of almost all the citrus fruits. Only the peel of pomelo and red grapefruit contained about 20% more potassium than the pulp (Table 2). Research by Barros et al. [11], however, indicates that the peel of orange, lime, and lemon accumulates more potassium than the pulp.

In addition to potassium, sodium is also responsible for regulation of the water and electrolyte balance. There is much less sodium than potassium in citrus fruits, which is important information for those who have problems with blood pressure regulation and conditions associated with hypertension [17]. Lime pulp (Table 2), for example, had significantly the highest content of sodium, and a lime weighing about 100 g would provide only 0.2% of the DRI [20].

Comparison of sodium content between the pulp and peel of citrus fruits showed that except for mandarin and lemon, sodium content was significantly higher in the peel than in the flesh (Table 2). This was in line with a study by Barros et al. [11], which indicated significantly higher sodium content in the peel of orange, lime, and mandarin relative to their pulp. The greatest difference between pulp and peel was observed in pomelo and orange, as the peel of these two fruits was nearly seven and five times richer in this macronutrient than the pulp (Table 2).

Citrus fruits, in comparison with other fruits, such as apples, pears, melons, peaches, plums, mangoes, and bananas, are a valuable source of calcium, which plays an important role in building hard, strong bones [17]. Our study indicated that the pulp of the citrus fruits analyzed (pulp from one piece of fruit) provides about 1.5% (a mandarin weighing about 65 g or a lemon weighing about 80 g) to about 7.5% (a pomelo weighing about 600 g) of the DRI, as does the peel [20]. The calcium content in all the citrus fruits was more than 50% higher (*p* ≤ 0.05) in the peel than in the pulp, and the difference in pomelo was as high as 100% (Table 2). Similar observations have been made by Barros et al. [11], who also found that the calcium content in the peel of Tahiti lime and Sweet lime was significantly higher than in the pulp, which contained only trace amounts. Therefore, the removal of this part of the fruit greatly reduces its nutritional value and also allows this element to enter the environment. Research has shown that calcium of vegetable origin is well absorbed from the human digestive tract [21], so losses of it should be limited by using citrus peels for consumption.

Citrus fruits are also a valuable source of phosphorus, which together with calcium, participates in the formation of strong bones and teeth [22]. As in the case of calcium, the citrus fruit peels had a higher concentration of this element than the pulp, but statistical significance was noted only in the case of lemon and red grapefruit (the difference between the peel and the pulp was about 32%) (Table 2). It should be noted, however, that the pulp from one pomelo fruit (about 600 g) provides from 9 to 16% of the DRI for phosphorus, which is especially important in the diet of young people and pregnant and lactating women. The rind of one pomelo fruit (about 320 g) would provide about 30–40% less of the DRI for phosphorus than the pulp [20]. However, like foods derived from plant seeds (e.g., beans, peas, cereals, and nuts), fruits contain phytic acid (also called phytate), a stored form of phosphorus that is not directly available to humans [20]. We found no information in the available literature on the phosphorus content of individual parts of citrus fruits. This information is extremely important because phosphorus, potassium and nitrogen are the elements with the greatest influence on fruit characteristics [23].

Analysis of the magnesium content in individual parts of the fruits revealed that the peel had higher content of this macronutrient than the pulp, with significant differences noted in the case of orange, pomelo, lemon, and red and white grapefruit (Table 2). This was consistent with results obtained by Barros et al. [11], which showed higher magnesium content in orange, lime, and mandarin peel compared with their flesh. The greatest difference between the pulp and the peel was found in the lemon, whose peel accumulated about 37% more magnesium than the pulp (Table 2). The smallest difference between the pulp and the peel was observed in the lime, in which only about 12% more magnesium was accumulated in the peel than in the flesh (*p* = 0.366) (Table 2). Barros et al. [11] found that magnesium content was more than four times higher in the peel of lime than in the pulp.

The dominant micronutrient in both the peel and the pulp of the analyzed citrus fruits was iron (Table 3). Its concentration in the peel was significantly higher than in the pulp in all varieties of citrus fruit (Table 3), which is supported by studies by Barros et al. [11] and Gorinstein et al. [24]. The greatest difference was noted in the lime, in which iron content was about twice as high in the peel. In one lime cultivar, Barros et al. [11] recorded iron content as much as six times higher in the peel than in the flesh. The smallest difference between the peel and the pulp was found in pomelo, at about 13% (Table 3). It should be noted, however, that pomelo pulp contained significantly more of this micronutrient than the other fruits. This is very valuable information, especially for people with high iron requirements, i.e., women of childbearing age (pulp from one of the analyzed pomelos would provide about 15% of the DRI, while the peel would provide about 7% of the DRI) and pregnant women (pulp from one of the analyzed pomelos would provide about 10% of the DRI, while the peel would provide about 5% of the DRI).

An equally important micronutrient is zinc, which protects the body against oxidative stress and stimulates immune mechanisms [25]. Its content in the peel of orange, lemon, and all grapefruit varieties was significantly higher than in the pulp (Table 3). These dependencies are supported by the research of Barros et al. [11] and Gorinstein et al. [24]. The greatest difference in zinc content between the peel and the pulp (90%) was recorded in the red grapefruit (*p* = 0.002).

Manganese is present in much smaller amounts in citrus fruits. Its quantity in 100 g of the fruits tested would provide only 1–2% of the DRI [20]. Its quantity in the flesh of nearly all the citrus fruits (except lemon) was significantly lower than in the peel (Table 3). The greatest difference was recorded in the pomelo, whose peel contained ten times more manganese than its pulp (Table 3). Substantial differences in manganese content between the peel and the pulp have been reported by Barros et al. [11]. Consumption of the pulp of one pomelo fruit (about 600 g) would provide on average about 30% of the DRI of magnesium, which is particularly important in the diet of older people suffering from depression, insomnia, or recurrent muscle cramps, as well as in physically active individuals [26].

The elements found in trace amounts in most fruits include selenium. Selenium strengthens the immune system, but in amounts exceeding the daily allowable intake, it is toxic to humans [27]. According to a study by Turner and Burri [1], citrus fruits are rich in this element. Comparison of the selenium content between the pulp and the peel of all tested citrus fruits showed that the amount of selenium was significantly higher in the peels than in the pulp (Table 3). The greatest difference in selenium content in the peel vs. the pulp was found in the lemon, in which the peel accumulated more than twice as much selenium as the pulp (Table 3).

### Mineral Elements in Whole Citrus Fruits

Our study indicated that the highest potassium content among the tested fruits was observed in lime and orange (Table 4), which is consistent with studies by Baghurst et al. [17] and Liu et al. [28]. The results can be helpful in establishing dietary guidelines for people suffering from potassium deficiency, as well as for lactating women, whose potassium requirement is higher, at about 5.1 g/d [20]. The whole fruit of pomelo, lemon, and red grapefruit was the least rich in this macroelement (Table 4).

Of the citrus samples studied, lime was clearly the richest in sodium (Table 4), which is confirmed by Baghurst et al. [17]. The whole lemon and mandarin contained less than half the quantity noted in the lime. It should be noted that in the other whole citrus fruits, the amount of sodium did not exceed 1 mg 100 g^−1^, with the lowest sodium content found in grapefruit, irrespective of the variety (Table 4). Research by Paul and Shaha [29] has confirmed that citrus fruits, i.e., oranges and pomelos, are not a rich source of sodium, so they can be used in diets for people with cardiac or kidney problems or those susceptible to osteoporosis [19, 30, 31]. Among the citrus fruits analyzed, the lime had the highest concentration of calcium, nearly 2.5 times higher than in the pomelo, nearly twice as high as in the mandarin, lemon, and all grapefruit varieties, and almost 1.5 times as high as in the orange (*p* ≤ 0.05) (Table 4). Research by Baghurst et al. [17] and Liu et al. [32] indicates that the orange is the citrus fruit with the highest calcium concentration. However, in a study by Paul and Shaha [29], lemon was the most calcium-rich citrus fruit. In the present study, the pomelo had the lowest calcium content (Table 4), as in the research by Paul and Shaha [29].

The orange was the citrus fruit with the highest phosphorus content (Table 4). This was in line with research by Baghurst et al. [17] and Paul and Shaha [29]. No significant differences in phosphorus content were observed between the other citrus fruit species (pomelo, mandarin, lemon, lime, or red, green and white grapefruit) (Table 4). Baghurst et al. [17] also observed no significant differences in phosphorus content between lemon, mandarin, grapefruit, and lime, and Paul and Shaha [29] reported no difference between grapefruit and pomelo.

The citrus fruit with the highest magnesium content was pomelo (*p* ≤ 0.05) (Table 4). This was in line with the research of Paul and Shaha [29]. It is worth noting that the pomelo fruits had the highest content of this macronutrient in both the pulp and the peel (Table 2).

No significant differences in magnesium content were observed between the other citrus species (orange, mandarin, lemon, lime, red grapefruit, green grapefruit, and white grapefruit), and the results were 24.59 ± 17.49 mg 100 g^−1^ (Table 4). The fruits with the highest magnesium content are the mandarin and orange according to Baghurst et al. [17] and Caengprasath et al. [33], and the lime according to Baghurst et al. [17]. These discrepancies in results may be due to factors such as the type of soil the fruit was grown on Aular et al. [23].

Our study indicated that the pomelo was the richest in iron (Table 4). Similar relationships were observed by Paul and Shaha [29]. Therefore, pomelo is recommended by physicians and dieticians to fight anemia, as well as oxidative stress, bacteria, and viruses [33]. Oranges also had high iron concentrations, which is in line with results reported by Baghurst et al. [17]. Grapefruit had less than half that amount of iron, irrespective of the cultivar (Table 4). A similar relationship was noted by Baghurst et al. [17] and Liu et al. [32], who also identified grapefruit as the citrus fruit with the least iron. It should be noted that only 1–5 percentage of total iron in fruits occurs is available to humans and strongly influenced by the presence of other food components [34]. Therefore, according to Barros et al. [11], the citrus fruit cannot be classified as “rich in” or a “source of” iron. The availability of this element is significantly affected, for example, by the presence of ascorbic acid [11].

The red and green grapefruit, lime, and mandarin contained significantly more zinc than the other citrus fruits (Table 4). However, in studies by Paul and Shaha [29] and Baghurst et al. [17], oranges were the citrus fruit with the highest content of this element. The pomelo was the least rich in zinc, with content less than half that of the other citrus fruits, in both the pulp and the peel (Tables 3, 4).

The pomelo and orange were also the fruits with the highest copper content (Table 4). A 100 g portion of these fruits would provide about 4–5% of the DRI of this element for adults [20]. It should be noted that of the total amount of this micronutrient in the pomelo, as much as 80% was contained in the peel (Tables 3, 4). The lowest content of this element was noted in lemon, mandarin, and lime (Table 4). Baghurst et al. [17] found lime to be the citrus fruit richest in copper. However, Paul and Shaha [29] indicated lemon as the citrus fruit with the highest content of this element, whereas according to research by Liu et al. [32], orange and mandarin have the highest copper content.

Significantly higher content of manganese compared with the other fruits was noted in the pomelos, as well as the oranges and mandarins (Table 4). Similar findings were reported by Baghurst et al. [17], who found the highest manganese content in orange, mandarin, and lemon. According to Liu et al. [28], mandarin is the citrus fruit with the highest manganese content. The smallest content of this micronutrient was observed in lime; it was more than three times lower than in orange and pomelo (Table 4).

Thus the total content of this micronutrient in citrus fruits was influenced by its content in the peel (Table 4). Therefore, removing this part of the fruit greatly reduces its nutritional value and also allows this element to enter the environment. The best example of this is pomelo, in which 90% of the total manganese content was in the peel (Tables 3, 4).

Significantly (*p* ≤ 0.05) higher selenium concentrations were noted in lemon and green grapefruit among the analyzed fruits (Table 4), while the red grapefruit contained the smallest amount of this micronutrient (Table 4). Baghurst et al. [17] reported lime and grapefruit to be the citrus fruits most abundant in selenium.

## Summary and Conclusions

Both the pulp and the peel of citrus fruits are valuable sources of macro- and micronutrients. Their content in the peel of most of the fruits tested far exceeds their quantity in the pulp, and for this reason, special attention should be paid to its potential use as a component of a functional food (designer foods) or in the pharmaceutical industry. The peel of citrus fruits can be used to produce mineral preparations of varying composition and properties, or extruded breakfast cereals with a high content of selected minerals, such as potassium, phosphorus, and calcium.

With regard to nutrition and health, increased consumption of specific citrus fruits may help to meet the body’s ongoing demand for individual minerals. The research showed that oranges and pomelos contain the most iron and copper, so they could be recommended in cases such as hemoglobin production disorders resulting from a deficiency of these elements. Oranges can additionally enrich the body with potassium, phosphorus, and manganese, while lime can be a source of calcium, zinc, sodium, and especially potassium. It should also be noted that all citrus fruits are a very valuable source of potassium, which is needed to ensure the water and electrolyte balance.
